# SATI-Q Registry: 20 years of experience with quality benchmarking in intensive care units

**DOI:** 10.62675/2965-2774.20250033-2

**Published:** 2025-07-15

**Authors:** María del Pilar Arias López, Ariel Leonardo Fernandez, Antonio Gallesio, María Elena Ratto

**Affiliations:** 1 Hospital de Niños Ricardo Gutiérrez Pediatric Intensive Care Unit Buenos Aires Argentina Pediatric Intensive Care Unit, Hospital de Niños Ricardo Gutiérrez - Buenos Aires, Argentina.; 2 Sociedad Argentina de Terapia Intensiva Management, Quality and Data Committee Buenos Aires Argentina Management, Quality and Data Committee, Sociedad Argentina de Terapia Intensiva - Buenos Aires, Argentina.; 3 Hospital Italiano de Buenos Aires Intensive Care Unit Buenos Aires Argentina Intensive Care Unit, Hospital Italiano de Buenos Aires - Buenos Aires, Argentina.

## INTRODUCTION

Intensive care units (ICUs) concentrate patients with high clinical complexity who require advanced and costly diagnostic and treatment techniques along with significant resource utilization. The main objective of ICUs is to provide critically ill patients with safe, comprehensive, evidence-based and efficient care. Standardized benchmarking focused on monitoring quality indicators is essential for identifying and promoting opportunities for improvement.^(1,2)^

The first global quality benchmarking (QB) registries arose from the need to optimize ICU performance.^(3)^ In line with these initiatives, the SATI-Q Registry was established in Argentina in 2002, supported by the *Sociedad Argentina de Terapia Intensiva* (SATI). This manuscript outlines the evolution of the SATI-Q Registry, highlighting its achievements and challenges with respect to enhancing ICU performance across Argentina.

## THE SATI-Q REGISTRY

The SATI-Q Registry was created in Argentina on the basis of the National Programme for Quality Assurance in Medical Care as a voluntary participation programme. The goal was to generate a multicenter network of prospective and permanent registries of quality indicators.^(4,5)^ This framework enables ICUs to evaluate and compare their performance with other local or international units.^(6)^ The primary objective of the registry is to improve critical care patient care quality through initiatives informed by local data.

The SATI-Q includes ICUs nationwide and encompasses various levels of complexity and infrastructure. The participating units use the freely provided SATI-Q software to monitor predefined quality indicators established by the SATI Management Committee in a standardized format. Since 2019, ICUs have also been able to participate via an interoperable format.

Participating centers prospectively record admission sources and diagnoses of patients admitted to the ICU and pediatric ICU, severity of illness (APACHE II and Pediatric Index of Mortality [PIM] 3), intensity of care received (therapeutic intervention scoring system [TISS] score), the use of invasive devices and risk-adjusted outcomes (vital status at discharge). The following quality indicators form the basis for the QB reports: rates of device-associated infections (ventilator-associated pneumonia, central line-associated bloodstream infection, and catheter-associated urinary tract infection), surgical site infection, self-extubations, pressure ulcer rates, fall rates, and accidental nasogastric tube dislodgement rates.

The data are submitted annually in encrypted, anonymized form via SATI-Q software or through interoperable formats such as electronic data interchange documents (EDSs) or fast health care interoperability resources (FHIRs) in accordance with health level (HL) 7 standards.^(7)^

The data collected are then used to create a consolidated database, thus producing publicly available annual general reports and confidential comparative reports for each unit and enabling ICUs to benchmark their performance against other participating units.^(6)^

## SATI-Q REPORTS

**Figure 1:**
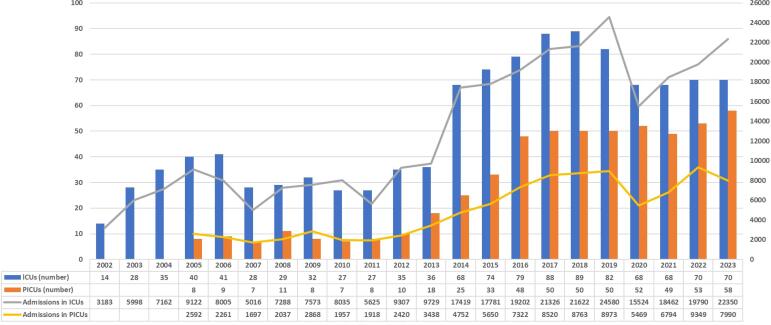
Number of intensive care units, pediatric intensive care units and admissions from 2003 to 2023.

The first QB report was published in 2003 and was based on data recorded by 28 adult ICUs. In 2005, the first report for pediatric ICUs was published. Currently, 128 adult and pediatric ICUs participate in the programme, representing institutions across Argentina with public, private and social security funding. Figure 1 shows the number of adult ICUs, pediatric ICUs and annual records analyzed since 2003, highlighting the expansion of the registry and its database growth. The registry currently comprises more than 284,000 deidentified records of hospitalizations in adult ICUs and 94,770 in pediatric ICUs in Argentina. The latest report (2023) analyzed 22,350 adult ICU discharges and 7,990 pediatric ICU discharges. The participating units and their locations are described in tables 1S and 2S and figure 1S (Supplementary Material); the 2023 reports are also detailed in the Supplementary Material.

ICU - intensive care unit; PICU - pediatric intensive care unit.

In line with the Programme's data policy, annual general reports are publicly accessible on the SATI-Q website,^(8)^ allowing users to track the evolution of monitored indicators.

Additionally, since 2019, the SATI-Q has been a member of the LOGIC Consortium (Linking of Global Intensive Care), an international QB network that provides annually updated aggregate data from the National Registries and networks that make up the consortium.^(9)^ Thus, ICUs that participate in the SATI-Q registry can compare their performance with international standards.

Figures 2S to 9S (Supplementary Material) present trends in the key SATI-Q quality indicators over time. The main patient's characteristics and outcomes in the ICUs included in the last LOGIC report (2023) compared with the SATI-Q data are presented in table 3S (Supplementary Material).

## SATI-Q: ACHIEVEMENTS AND CHALLENGES

The information provided by the QB reports enables institutions to benchmark their performance against national and international standards. With data representing ICUs from diverse regions, complexities and funding, the reports offer a comprehensive overview of intensive care medicine in Argentina. Participation allows ICUs to assess their results in real time, supporting local improvement initiatives. Over time, these reports have highlighted key trends, such as reduced infection rates and declining pediatric ICU mortality. Although changes in critical care outcomes are likely to be multifactorial and heterogeneous, participation in multicenter QB programmes is recommended as a highly effective strategy for driving improvements in critical care.^(10)^

Despite these recognized benefits, the SATI-Q is a voluntary registry, and thus, participation can easily be compromised by human and technologic resource constraints. An example of these constraints is the decrease in the number of ICUs and patients included during 2020 due to the COVID-19 pandemic, when the health care system was overloaded (Figure 1).

In addition to monitoring quality indicators, the SATI-Q has laid the foundation for a collaborative, voluntary data collection network that supports clinical research to increase critical care quality. Successful experiences of this potential include multicenter pediatric validation studies of the Pediatric Index of Mortality (PIM 2 and PIM 3) risk score in Argentina and later in Latin America, among others.^(11–14)^

SATI-Q also collaborates with leading groups such as the Massachusetts Institute of Technology's Computational Physiology Laboratory. As part of the Global Open-Source Severity Illness Score (GOSSIS) Consortium, the SATI-Q contributes to the development of prognostic scores for adult ICU mortality.^(15)^

With a database of more than 240,000 ICU admission records, applying artificial intelligence to the SATI-Q Registry presents significant opportunities to enhance critical care. AI-powered analysis can uncover hidden patterns, predict outcomes, and identify key risk factors. Machine learning algorithms have the potential to refine prognostic models and optimize resource allocation, such as accurately predicting length of stay, ultimately improving the quality, efficiency, and effectiveness of critical care delivery.

## CONCLUSIONS

The SATI-Q is a consolidated registry that was established more than 20 years ago. This registry generates quality benchmarking reports, thus enabling intensive care units to assess their performance over time and compare it with other local and international institutions. The registry has also fostered a collaborative research network with the potential to support epidemiological studies to advance the care of critically ill adult and pediatric patients. Efforts are currently focused on enhancing the interoperability of SATI-Q software to facilitate integration with the national health information system, maximizing data quality and the use of information for decision-making and management. Undoubtedly, the development and maintenance of an intensive care unit inpatient registry provides valuable information for improving the quality of care and constitutes an open challenge for critical care teams at both the local and regional levels.
