# Supplementary material for: SATI-Q Registry: 20 years of experience with quality benchmarking in intensive care units
Source: Crit Care Sci. 2025 Jul 15;37:20250033-2. doi: 10.62675/2965-2774.20250033-2 (PMC12040418; doi:10.62675/2965-2774.20250033-2)
Supplement: Supplementary file 1 [file 2965-2774-ccsci-37-e20250033-Suppl01.pdf]

Critical Care Science
https://doi.org/10.62675/2965-2774.20250033-2

SUPPLEMENTARY MATERIAL

SATI-Q Registry: 20 years of experience in quality benchmarking in intensive care units

María del Pilar Arias López[1,2], Ariel Leonardo Fernandez[2], Antonio Gallesio[2,3], María Elena Ratto[2]

2023 SATI-Q REPORT

SATI-Q Report is based on data recorded by the following intensive care units:

Table 1S - Participant intensive care units

Intensive care units	Geographic location	ICUs' characteristics
Área Programa General Roca - Hospital Francisco López Lima	Río Negro	Public
Clínica de Imágenes	Neuquén	Private
Clínica del Valle SRL	Chubut	Private
Clínica Modelo de Entre Ríos	Entre Ríos	Private
Clínica Parque	Entre Ríos	Private
Clínica Pasteur	Neuquén	Private
Clínica San Agustín	Río Negro	Private
Clínica San Jorge	Tierra del Fuego	Private
Clínica Universitaria "Reina Fabiola"	Córdoba	Private
Hospital Aeronáutico	Ciudad Autónoma de Buenos Aires	Social Security
Hospital Área Programa Dr Pedro Moguillansky	Río Negro	Public
Hospital Castro Rendón	Neuquén	Public
Hospital Centeno	La Pampa	Public
Hospital Central de Mendoza	Mendoza	Public
Hospital Central Ramón Carrillo (San Luis)	San Luis	Public
Hospital de Clínicas José de San Martín	Ciudad Autónoma de Buenos Aires	Public
Hospital de Emergencias Dr Clemente Álvarez	Santa Fe	Public
Hospital Dr Carlos Saporiti	Mendoza	Public
Hospital Dr Jaime Ferre	Santa Fe	Public
Hospital Español de Mendoza	Mendoza	Private
Hospital Eva Perón	Santa Fe	Public
Hospital General de Agudos Dr Carlos G Durand (UCI 1)	Ciudad Autónoma de Buenos Aires	Public
Hospital General de Agudos Dr Carlos G Durand (UCI 2)	Ciudad Autónoma de Buenos Aires	Public
Hospital Interzonal de Agudos Vicente López y Planes	Buenos Aires	Public
Hospital Interzonal Zapala Dr Juan Jorge Pose	Neuquén	Public
Hospital Italiano de Córdoba	Córdoba	Private
Hospital Iturraspe	Santa Fe	Public

Continue...

This is an open access article under the CC BY license (https://creativecommons.org/licenses/by/4.0/).

...continuation

Hospital José María Cullen	Santa Fe	Public
Hospital Justo José de Urquiza	Entre Ríos	Public
Hospital Madre Catalina Rodriguez	San Luis	Public
Hospital Militar de Paraná	Entre Ríos	Public
Hospital Municipal Dr Carlos Macías	Buenos Aires	Public
Hospital Municipal Dr. Bernardo Houssay	Buenos Aires	Public
Hospital Municipal Príncipe de Asturias	Córdoba	Public
Hospital Naval Pedro Mallo	Ciudad Autónoma de Buenos Aires	Social Security
Hospital Privado Centro Médico de Córdoba	Córdoba	Private
Hospital Privado de Rosario	Santa Fe	Private
Hospital Provincial de Rosario – UTI A	Santa Fe	Public
Hospital Provincial Ramon Carrillo	Neuquén	Public
Hospital Regional de Comodoro Rivadavia	Chubut	Public
Hospital Regional Dr Jose A Ceballos Bell Ville	Córdoba	Public
Hospital SAMIC El Calafate	Santa Cruz	Public
Hospital Sofía Terrero Santamarina	Buenos Aires	Public
Hospital Zonal Esquel	Chubut	Public
Instituto Alexander Fleming	Ciudad Autónoma de Buenos Aires	Private
Instituto de Trasplante y Alta Complejidad	Ciudad Autónoma de Buenos Aires	Private
Instituto Quirúrgico del Callao	Buenos Aires	Private
Maternidad Provincial Doctora Teresita Baigorria	San Luis	Public
Nuevo Hospital Río Cuarto "San Antonio de Padua"	Cordoba	Public
Policlínico Modelo de Cipolletti S.A.	Río Negro	Private
Policlínico PAMI 1	Santa Fe	Social Security
Policlinico Regional Juan D. Perón de Villa Mercedes	San Luis	Private
Sanatorio Adventista del Plata	Entre Ríos	Private
Sanatorio Aliare	Santa Fe	Private
Sanatorio Anchorena Itoiz	Ciudad Autónoma de Buenos Aires	Private
Sanatorio Británico de Rosario	Santa Fe	Private
Sanatorio del Oeste	Buenos Aires	Private
Sanatorio Diagnóstico	Santa Fe	Private
Sanatorio Fueguino de Diagnóstico y Tratamiento	Tierra del Fuego	Private
Sanatorio Garay (UCI A)	Santa Fe	Private
Sanatorio La entrerriana	Entre Ríos	Private
Sanatorio Las Lomas	Buenos Aires	Private
Sanatorio Mater Dei	Ciudad Autónoma de Buenos Aires	Private
Sanatorio Nosti	Santa Fe	Private
Sanatorio Privado San Gerónimo	Santa Fe	Private
Sanatorio San Lucas	Buenos Aires	Private

According to the Argentine Ministry of Health the number of intensive care unit beds in the period was 9419. A total of 846 were included in SATI-Q report (9%).

Table 2S - Participant pediatric intensive care units

Pediatric intensive care units	Geographic location	Pediatric ICUs' characteristics
Alexander Fleming	Mendoza	Private - General hospital
Clinica del Niño Quilmes	Buenos Aires	Private - General hospital
Clínica Modelo de Morón	Buenos Aires	Private - General hospital
Clínica Universitaria "Reina Fabiola"	Córdoba	Private - General hospital
Complejo Médico de la Policía Federal Argentina Churruca Visca	Ciudad Autónoma de Buenos Aires	Social Security - General hospital
Fundación Hospitalaria	Ciudad Autónoma de Buenos Aires	Private - General hospital
Hospital Cuenca Alta Nestor Kirschner	Buenos Aires	Public - General hospital
Hospital de Niños Sor María Ludovica de La Plata/Cardiovascular	Buenos Aires	Public - Pediatric hospital
Hospital de Niños Dr. Héctor Quintana	Jujuy	Public - Pediatric hospital
Hospital de Niños Dr. R. Gutiérrez	Ciudad Autónoma de Buenos Aires	Public - Pediatric hospital
Hospital de Niños Santísima Trinidad Córdoba	Córdoba	Public - Pediatric hospital
Hospital de Niños Sor María Ludovica La Plata	Buenos Aires	Public - Pediatric hospital
Hospital de Niños V. J. Vilela Cardiovascular	Santa Fe	Public -Pediatric hospital
Hospital de Niños Zona Norte	Santa Fe	Public - Pediatric hospital
Hospital del Niño Jesús de Tucumán	Tucumán	Public - Pediatric hospital
Hospital del Niño Jesús de Tucumán UCO	Tucumán	Public - Pediatric hospital
Hospital Dr Humberto Notti	Mendoza	Public - Pediatric hospital
Hospital Dr Humberto Notti/ Cardiovascular	Mendoza	Public - Pediatric hospital
Hospital Dr O Alassia	Santa Fe	Public - Pediatric hospital
Hospital El Cruce "Néstor Kirchner"	Buenos Aires	Public - General hospital
Hospital Español de Rosario	Santa Fe	Public - General hospital
Hospital Francisco López Lima	Río Negro	Public - General hospital
Hospital General de Agudos "Carlos G Durand"	Ciudad Autónoma de Buenos Aires	Public - General hospital
Hospital General de Niños "Pedro de Elizalde"	Ciudad Autónoma de Buenos Aires	Public - General hospital
Hospital General de Niños "Pedro de Elizalde" UCIP B	Ciudad Autónoma de Buenos Aires	Public - Pediatric hospital
Hospital Guillermo Rawson	San Juan	Public - Pediatric hospital
Hospital Infantil de Córdoba	Córdoba	Public - Pediatric hospital
Hospital Materno Infantil de Salta	Salta	Public - Pediatric hospital
Hospital Municipal de Quemados	Ciudad Autónoma de Buenos Aires	Public - General hospital
Hospital Municipal Federico Falcón	Buenos Aires	Public - General hospital
Hospital Pediátrico "Dr. Claudio Zin"	Buenos Aires	Public - Pediatric hospital
Hospital Pediátrico del Niño Jesús	Córdoba	Public - Pediatric hospital
Hospital Pediátrico San Luis	San Luis	Public - Pediatric hospital
Hospital Provincial de Neuquen - Terapia Intermedia	Neuquén	Public - General hospital
Hospital Provincial de Rosario	Santa Fe	Public - General hospital
Hospital Regional Castro Rendón	Neuquén	Public - General hospital
Hospital Regional Louis Pasteur	Cordoba	Public - General hospital
Hospital Universitario Austral	Buenos Aires	Private - General hospital
Hospital Zonal "Doctor Ramón Carrillo"	Río Negro	Public - General hospital

Continue...

...continuation

Hospital Zonal de Trelew "Adolfo Margara"	Chubut	Public - General hospital
Policlinico Regional Villa Mercedes	San Luis	Public - General hospital
Sanatorio Anchorena	Ciudad Autónoma de Buenos Aires	Private - General hospital
Sanatorio Anchorena - San Martin	Buenos Aires	Private - General hospital
Sanatorio Argentino	San Juan	Private - General hospital
Sanatorio de la Trinidad Mitre	Ciudad Autónoma de Buenos Aires	Private - General hospital
Sanatorio de la Trinidad Ramos Mejía	Buenos Aires	Private - General hospital
Sanatorio de Niños	Santa Fe	Private - General hospital

According to the Argentine Ministry of Health the number of pediatric intensive care unit beds in the period was 1293. A total of 496 were included in the 2023 SATI-Q pediatric report (38.3%).

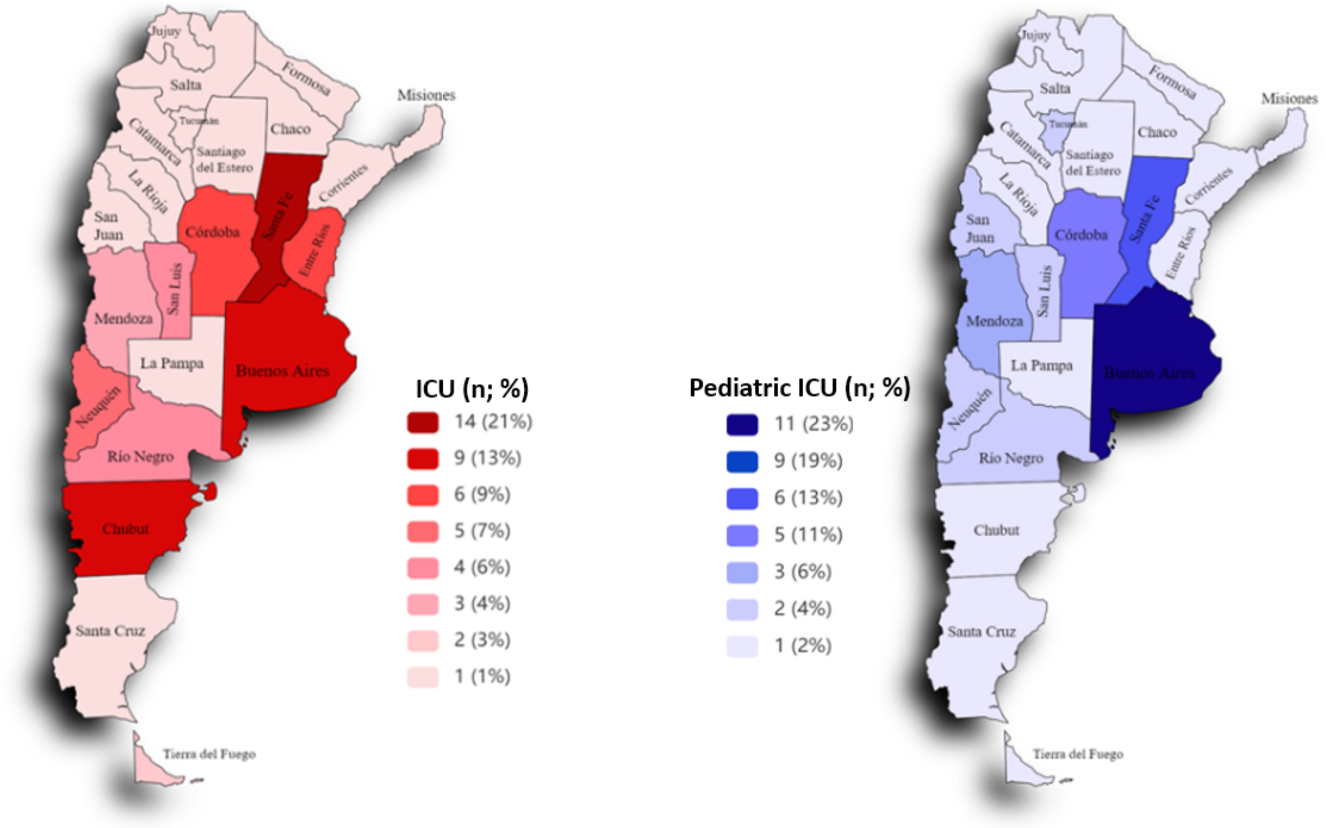

ICU - intensive care unit.

Figure 1S - Geographical distribution of intensive care units and pediatric intensive care units that participated in SATI-Q reports.

ICU Report 2023

SATI-Q Report

From: 01/01/2023 **To:** 31/12/2023 **Admissions:** 22350 **Patients:** 20694

Lenght of stay (days): Min: 1 **Max:** 338 **Mean:** 6,68 **Median:** 3,00 **SD:** 12,61

Observed mortality: 3469 15,52% **Predicted mortality:** 19,44% **(SMR:** 0,80**)**

Patients days: 149293

**Admissions with:**	**TOTAL**	**%**
Sores	381	1,70
Noninvasive mechanical ventilation (NIV)	563	2,52
High flow nasal cannula (HFNC)	542	2,43
Invasive mechanical ventilation (IMV)	5716	25,57
Ventilator associated pneumonia (VAP)	821	14,36
Central venous catheter (CVC)	7017	31,40
Central line associated bloodstream infections (CLABSI)	199	2,84
Urinary catheter	12545	56,13
Catheter associated urinary tract infection (CAUTI)	297	2,37
Falls	31	0,14
Readmissions	710	3,18
Gender femenine	8496	38,01
Gender male	13854	61,99

	**Mode**	**Median**	**Total**	**%**	**Min**	**Máx**	**Mean**	**SD**
APACHE score	10,00	12,00			1,00	56,00	13,57	8,20
Therapeutic intervention scoring system-28	15,00	21,00			1,00	60,00	21,63	7,91
Age	64,00	61,00			16,00	103,00	57,83	18,54
Invasive mechanical ventilation days	1,00	5,00	58.572	39,23	1,00	188,00	10,34	15,13
Noninvasive mechanical ventilation days	1,00	2,00	1.950	1,31	1,00	114,00	3,49	6,13
HFNC Days	1,00	3,00	2.058	1,38	1,00	49,00	3,87	4,14
CVC Days	1,00	5,00	71.622	47,97	1,00	310,00	10,21	15,59
Urinary catheter days	1,00	3,00	93.843	62,86	1,00	310,00	7,48	12,26

VAP rate (per 1000 IMV days)	14,02
CLABSI (per 1000 CVC days)	2,78
CAUTI (per 1000 urinary catheter days)	3,16

Pediatric ICU's Report 2023

SATI-Q Report

From: 01/01/2023 **To:** 31/12/2023 **Admissions:** 7990 **Patients:** 7238

Lenght of stay (days): Min: 1 **Max.:** 338 **Mean:** 9,54 **Median:** 5,00 **SD:** 16,96

Pediatric ICU observed mortality: 334 4,18% **Predicted mortality:** 3,80% **(SMR:** 1,10**)**

Patient´s days: 76251

**Admissions with:**	**TOTAL**	**%**
Sores	69	0,86
Noninvasive mechanical ventilation (NIV)	1393	17,43
High flow nasal cannula (HFNC)	993	12,43
Invasive mechanical ventilation (IMV)	3868	48,41
Ventilator associated pneumonia (VAP)	242	6,26
Central venous catheter (CVC)	3781	47,32
Central line associated bloodstream infections (CLABSI)	118	3,12
Urinary catheter	3943	49,35
Catheter associated urinary tract infection (CAUTI)	150	3,80
Falls	3	0,04
Readmissions	188	2,35
Gender femenine	3285	41,11
Gender male	4705	58,89

	**Mode**	**Median**	**Total**	**%**	**Min**	**Máx**	**Mean**	**SD**
APACHE score	-4,93	-4,48			-8,63	40,38	-4,36	1,91
		1,12			0,02	99,99	3,80	9,33
Therapeutic intervention scoring system-28	16,00	21,00			1,00	61,00	22,36	7,96
Age	1,00	34,00			1,00	190,00	54,89	55,47
Invasive mechanical ventilation days	1,00	6,00	37.726	49,48	1,00	271,00	9,84	15,45
Noninvasive mechanical ventilation days	1,00	2,00	4.426	5,80	1,00	62,00	3,20	4,20
HFNC Days	1,00	3,00	3.731	4,89	1,00	121,00	3,77	5,60
CVC Days	6,00	7,00	43.145	56,58	1,00	232,00	11,41	14,67
Urinary catheter days	1,00	5,00	32.760	42,96	1,00	201,00	8,31	10,82

VAP rate (per 1000 IMV days)	6,41
CLABSI (per 1000 CVC days)	2,73
CAUTI (per 1000 urinary catheter days)	4,58

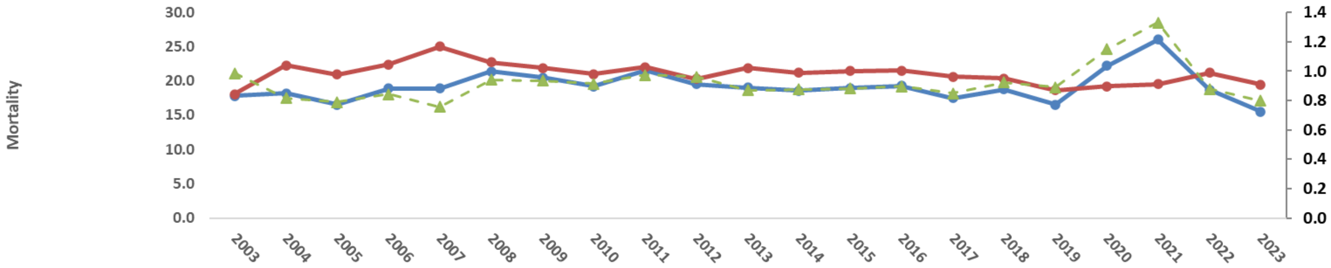

	2003	2004	2005	2006	2007	2008	2009	2010	2011	2012	2013	2014	2015	2016	2017	2018	2019	2020	2021	2022	2023
Observed Mortality%	17.8	18.2	16.5	18.9	18.9	21.4	20.5	19.2	21.5	19.5	19.0	18.6	18.9	19.3	17.5	18.8	16.5	22.2	26.0	18.7	15.52
Predicted Mortality %	18.0	22.3	21.0	22.4	25.0	22.7	21.9	21.0	22.0	20.3	21.9	21.2	21.5	21.5	20.6	20.3	18.7	19.2	19.5	21.2	19.44
SMR	1.0	0.8	0.8	0.8	0.8	0.9	0.9	0.9	1.0	1.0	0.9	0.9	0.9	0.9	0.9	0.9	0.9	1.2	1.3	0.9	0.8

SMR - standardized mortality rate.

Figure 2S - SATI-Q Registry: trends of observed mortality, predicted mortality and standardized mortality ratio in intensive care units.

Observed and predicted mortality are shown on the primary axis of the graph. The standardized mortality rate is shown on the secondary axis as a dotted line. Predicted mortality is calculated using APACHE II score. It's worth to note the increase in standardized mortality rate during years 2020 and 2021 due to the COVID-19 pandemic.

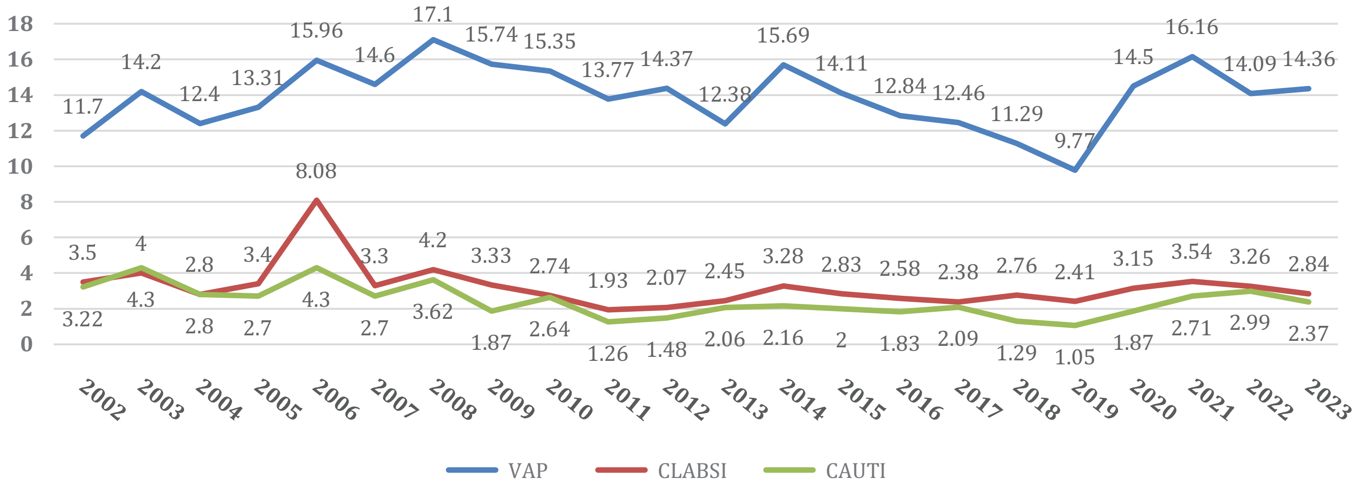

VAP - ventilator associated pneumonia; CLABSI - catheter associated bloodstream infections; CAUTI - catheter associated urinary tract infections.

Figure 3S - SATI-Q Registry: trends of device associated infection rates in intensive care units.

Device associated infection rates are expressed as incidence density (per 1000 days of device use).

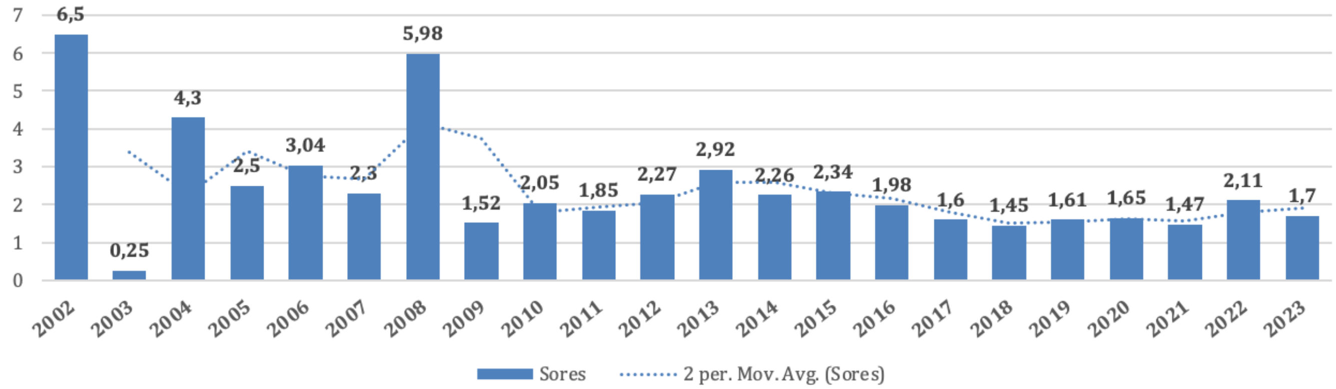

Figure 4S - SATI-Q Registry: trends of pressure ulcer-sores rates in intensive care units
Pressure ulcers - sore rates: proportion of patients that develop a pressure ulcer during their intensive care unit recovery.

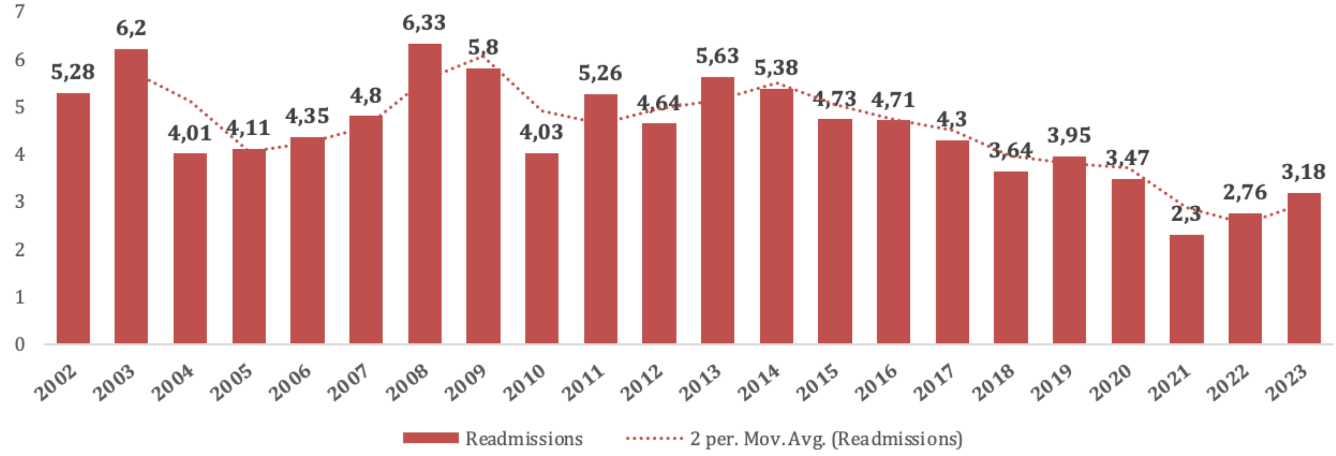

Figure 5S - SATI-Q Registry: trends of readmission rates in intensive care units
Readmission is defined as an unscheduled readmission that occurs within 48 hours of discharge from the intensive care unit.

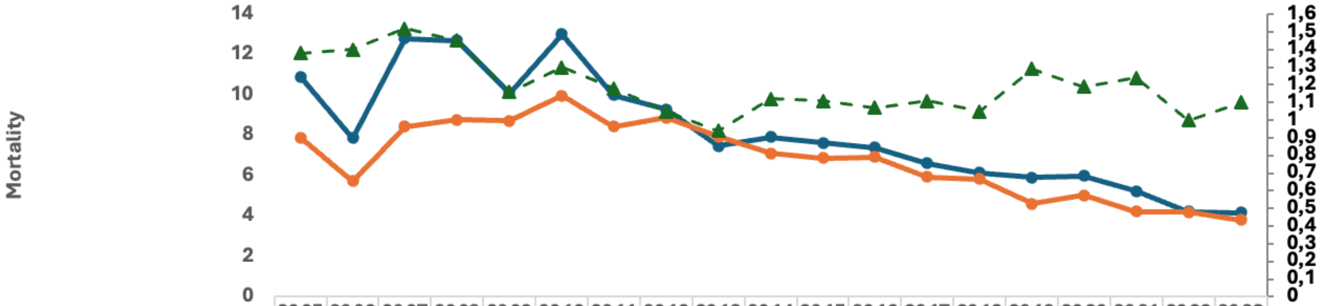

	2005	2006	2007	2008	2009	2010	2011	2012	2013	2014	2015	2016	2017	2018	2019	2020	2021	2022	2023
Mortality(%)	10,91	7,87	12,8	12,7	10,09	13	9,98	9,3	7,48	7,92	7,63	7,4	6,61	6,14	5,92	5,98	5,23	4,23	4,18
Expected mortality	7,88	5,74	8,44	8,77	8,72	9,97	8,43	8,87	7,94	7,1	6,87	6,92	5,94	5,84	4,6	5,03	4,23	4,2	3,8
SMR	1,38	1,4	1,52	1,45	1,16	1,3	1,18	1,05	0,94	1,12	1,11	1,07	1,11	1,05	1,29	1,19	1,24	1	1,1

SMR - standardized mortality rate.

Figure 6S - SATI-Q Registry: trends of observed mortality, predicted mortality and standardised mortality ratio in pediatric intensive care units.
Observed and predicted mortality are shown on the primary axis of the graph. The standardized mortality rate is shown on the secondary axis as a dotted line. Predicted mortality is calculated using Pediatric Index of Mortality 3 score since year 2019, previously Pediatric Index of Mortality 2 score was used. This modification explains the increase in standardized mortality rate observed in the year 2019. The impact of changing the prognostic score used to predict mortality was decided after performing the validation of Pediatric Index of Mortality 3 in a representative sample of patients admitted to Argentinean pediatric intensive care units.

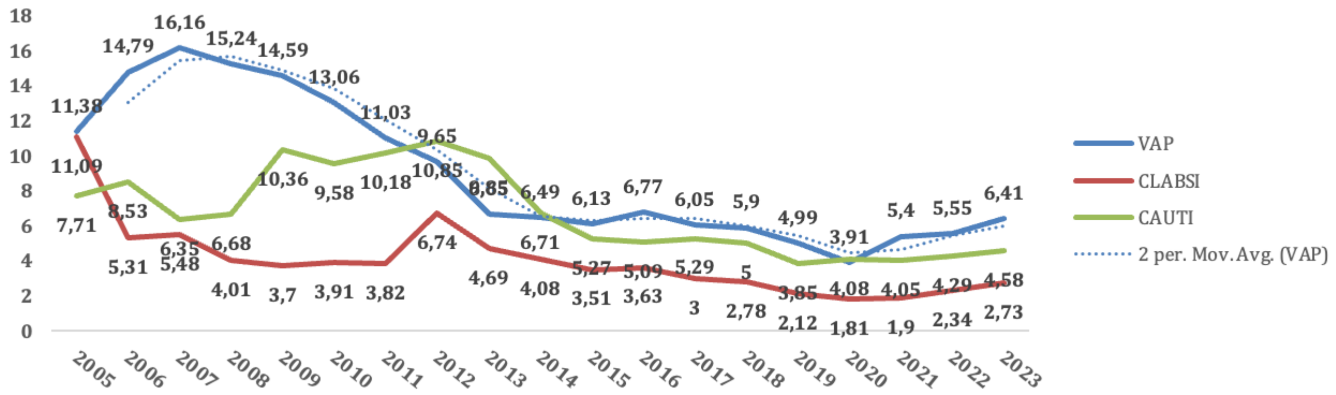

VAP - ventilator associated pneumonia; CLABSI - catheter associated bloodstream infections; CAUTI - catheter associated urinary tract infections.

Figure 7S - SATI-Q Registry: trends of device associated infections rates in pediatric intensive care units

Device associated infection rates are expressed as incidence density (per 1000 days of device use).

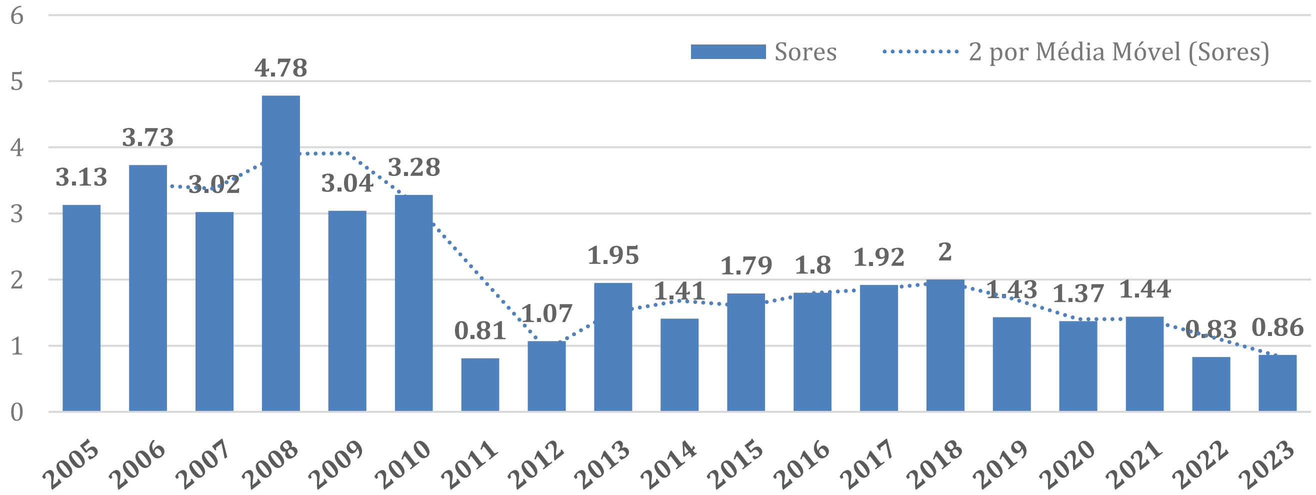

Figure 8S - SATI-Q Registry: trends of pressure ulcer-sore rates in pediatric intensive care units.

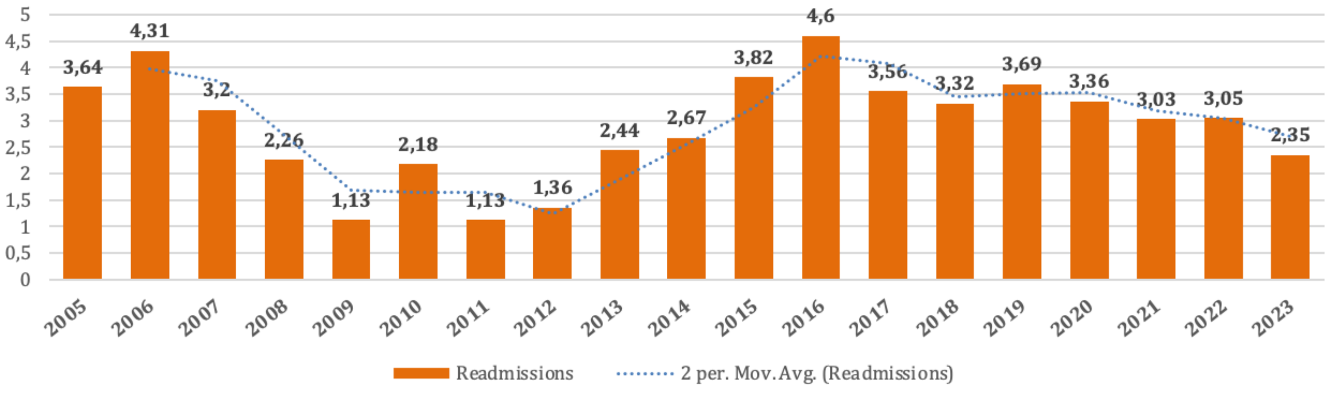

Figure 9S - SATI-Q Registry: trends of readmission rates device associated infections in pediatric intensive care units.

Readmission is defined as an unscheduled readmission that occurs within 48 hours of discharge from the pediatric intensive care unit.

Table 3S - Main patient's characteristics and outcomes in intensive care units that participate in Linking of Global Intensive Care consortium (Aggregated Data - Year 2023)

Registries	Country	Admissions (n)	Age (years; median)	Mechanical ventilation (n)	Mechanical ventilation (days; median)	ICU LOS (days; median)	ICU mortality (%)
ANZICS Australia	Australia	183,402	66.3	55,411	0.8	1.9	5.07
ANZICS New Zealand	New Zealand	16,487	62.8	6,804	0.8	1.8	7.73
Brazilian ICUs	Brazil	899,749	63.6	157,987	4	2.5	10.45
CCAA	Countries CCAA	19,471	46	5,063	2	3	16.57
IRIS India	India	21,097	56	5,026	1	3	9.18
NICE Netherlands	Netherlands	70,077	66	33,550	0.5	1.1	9.30
NICRF Nepal	Nepal	10,330	55	2,912	2	3	11.86
PRICE Pakistan	Pakistan	26,129	48	8,933	1	3	21.01
**SATI-Q Argentina**	**Argentina**	**22,350**	**57.8**	**5,716**	**5**	**3**	**15.52**
Uruguayan ICUs	Uruguay	5,594	59.9	2,533	3	2	18.48

Table created by the authors based on data available on the LOGIC platform. LOGIC Linking of Global Intensive Care. New international benchmarking; 2022. [Accessed 23 Feb 2025]. Available in https://icubenchmarking.com/new-international-benchmarking/. Includes 43 intensive care units from 21 hospitals of countries from Asia and Africa. SATI-Q aggregated data are highlighted in bold letters. Intensive care unit length of stay and days on mechanical ventilation are the highest compared to other registries or networks. ICU - intensive care unit; LOS - length of stay; ANZICS - Australian and New Zealand Intensive Care Society; CCAA - Critical Care Asia and Africa; IRIS - Indian Registry of Intensive Care Units; NICE - National Institute for Health and Care Excellence; NICRF - Nepal Intensive Care Research Foundation; PRICE - Pakistan Registry of Intensive CarE.
